# Fyn kinase: a key mechanistic regulator and therapeutic target in tauopathy and neurodegenerative diseases

**DOI:** 10.1186/s40035-026-00590-0

**Published:** 2026-10-01

**Authors:** Haiwen Wang, Xing Fan, Jianchao Ping, Qian Shao, Cai Wang, Hao Wang, Qiuhong Ji, Min Su, Wei Qian

**Affiliations:** 1 Department of Biochemistry and Molecular Biology, School of Medicine, Key Laboratory of Neuroregeneration of Jiangsu and Ministry of Education, Co-innovation Center of Neuroregeneration, Nantong University, Nantong, 226001 China; 2 Affiliated Hospital of Nantong University, Nantong, 226001 China

**Keywords:** Fyn kinase, Tauopathy, Neurodegenerative diseases, Neurotransmitters, Src family kinases

## Abstract

Synaptic dysfunction and intracellular accumulation of pathological tau are convergent hallmarks of Alzheimer’s disease (AD) and related tauopathies, yet the molecular mechanisms coupling these events to widespread neurodegeneration remain incompletely understood. The Src family kinase Fyn has emerged as a critical signaling hub that integrates upstream pathogenic triggers, such as amyloid-β (Aβ), with downstream neurotoxic cascades. Here, we review the multifaceted roles of Fyn in orchestrating synaptic failure, highlighting how Fyn recruitment by mislocalized tau promotes N-methyl-D-aspartate receptor hyperactivation and calcium dysregulation. We examine the biophysical basis of Fyn–tau interactions, particularly the role of liquid–liquid phase separation in stabilizing toxic signaling complexes. We also discuss how aberrant Fyn signaling in microglia and astrocytes amplifies neuroinflammation. Finally, we evaluate the potential of Fyn as a therapeutic target, emphasizing the necessity of strategies that selectively uncouple pathological signaling from physiological functions to effectively halt disease progression.

## Introduction

Abnormal hyperphosphorylation and mislocalization of the tau protein are not just isolated pathological events. They are phenomena of “systemic dysregulation” in various neurodegenerative diseases. Alterations of the conformation and interactions of tau can cause its transfer from the axonal microtubule network to the dendritic and synaptic regions. This leads to disruption of interregional signal transmission [1–3]. It should be noted that key pathological elements such as poor synaptic function, neuroinflammation, and neuronal loss often occur together and enhance each other. However, how these factors are interconnected and mutually amplified through common upstream molecular events, including disrupted synaptic activity, dysregulated calcium homeostasis, impaired protein homeostasis, and inflammatory cascades, remains to be elucidated.

Novel insights have been derived from studies of the “non-microtubule” functions of tau. An increasing body of evidence shows that tau is not just a cytoskeletal protein. Most of the tau proteins normally localize to axons, with a smaller amount found in the soma and dendrites [4–6]. For a prolonged period, the roles of tau were considered to be confined to establishing neuronal polarity, promoting axonal elongation, and facilitating transport as an axonal cytoskeletal protein [7–10]. The localization of tau in post-synapses implies a new role in postsynaptic signaling scaffolds and synaptic plasticity [3, 10–12]. External factors such as amyloid-β (Aβ), and intrinsic elements such as post-translational modification, lead to increased concentrations of tau in dendrites and post-synapses (mislocalization of tau) [10]. In Alzheimer’s disease (AD) and other tauopathies, the mislocalized dendritic tau plays a role in dendritic loss, abnormal postsynaptic activity, and cognitive dysfunction [1, 6, 13, 14]. As the earliest sign of neuronal malfunction in AD and related tauopathies, synaptic loss is also the strongest biological correlate of disease progression [6, 15–17].

The N-terminal region and the proline-rich region of tau are key to the synapse-related interactions. The proline-rich region can bind the Src homology (SH) 3 domains of signal molecules such as Fyn and Src, leading to phosphorylation of N-methyl-D-aspartate receptors (NMDARs). Abnormal tau phosphorylation disrupts these interactions, impairing downstream signal output [3, 18–20]. Therefore, the “toxicity” of tau pathology is manifested not merely in aggregation and tangle formation but, more importantly, in the signaling transduction pathway caused by its synaptic compartmentalization. This provides a direct molecular entry point for explaining early synaptic imbalance.

Among many candidate hub molecules, the status of the Src family non-receptor tyrosine kinase, Fyn, is particularly prominent. Fyn is located in the postsynaptic density (PSD). The SH3 domain of Fyn interacts with the Proline-X-X-Proline (PXXP) motifs located in the proline-rich region of tau [3, 10, 18, 21, 22]. In a tau-dependent manner, Fyn targets the post-synapse and binds to postsynaptic density protein 95 (PSD-95) (a key scaffolding protein for postsynaptic receptors) and NMDARs [3, 23]. The NMDAR subunit GluN2B, Fyn, tau, and PSD-95 form a complex at the synapse [3, 10, 23]. Studies on the effects of mutations in frontotemporal dementia and Parkinsonism associated with chromosome 17 (FTDP-17) on the Fyn–tau interaction suggest that aberrant interactions between tau and Fyn leads to neurodegenerative processes [10, 19, 24, 25].

Tau attracts Fyn to dendritic spines and Fyn directly phosphorylates tau at tyrosine residues (e.g., Tyr18). Moreover, pY18-tau is present in neurofibrillary tangles (NFTs) [3, 25]. The phosphorylation of GluN2B by Fyn at Y1472 potentiates the interaction between NMDAR and PSD-95 within synapses [3, 10, 22]. GluN2B phosphorylation by Fyn modulates the synaptic NMDAR content and enhances NMDAR-dependent synaptic currents [10, 22, 26–28]. Crucially, Ittner et al. [3] demonstrated that pathogenic tau variants (e.g., P301L) are enriched in dendritic spines and show more strong binding to Fyn, further enhancing NMDAR phosphorylation and its interaction with PSD-95. This suggests that tau pathology can amplify postsynaptic signaling via Fyn [3, 29]. Therefore, Fyn is not a bystander in tau pathology but a key amplifier that transforms tau mislocalization into toxic synaptic signals.

Fyn functions as a signaling hub, integrating Aβ and tau-mediated synaptic toxicity into one pathway. Amyloid-β oligomers (Aβo) activate Fyn through the cellular prion protein (PrP^C^)–metabotropic glutamate receptor 5 (mGluR5) complex, and then drive NMDAR phosphorylation and Ca^2+^ influx [30]. The interaction between Aβo and NMDARs disrupts Ca^2+^ balance, causing cell death related to oxidation and endoplasmic reticulum (ER) stress. Tau inhibits endoplasmic reticulum-associated degradation (ERAD), leading to the accumulation of proteins in the ER lumen, resulting in ER stress [31]. The ER stress further triggers the unfolded protein response (UPR). The core sensors of the UPR, namely PERK (protein kinase R-like endoplasmic reticulum kinase), IRE1 (Inositol-requiring enzyme 1), and ATF6 (Activating transcription factor 6), can be activated under multiple AD-related stresses, including Ca^2+^ imbalance, oxidative stress, proteasome inhibition, and metabolic/mitochondrial abnormalities. The UPR also influences synaptic plasticity and memory processes, making it a potential convergence point connecting “protein misfolding” and “synaptic failure” [32]. Taken together, the Aβo–PrP^C^–mGluR5–Fyn signaling disrupts the regulation of NMDAR containing GluN2B and Ca^2+^ homeostasis, whereas tau accumulation impairs ERAD and activates ER stress and the UPR. The two pathways may functionally converge on synaptic failure in AD [30, 31, 33–35].

The key role of Fyn has been demonstrated in genetics and disease models [30, 31]. In tauopathy mouse models, Fyn deletion significantly ameliorates behavioral deficits, attenuates neuroinflammation, restores synaptic protein levels, and reduces hyperphosphorylated tau and tau pathology [3, 36]. However, Fyn also plays a role in physiological long-term potentiation (LTP) and learning and memory. Global deletion may damage the physiological function [37, 38]. The challenge in developing Fyn as a therapeutic target resides in the selective inhibition of pathological signals by disrupting the pathogenic Aβ–Receptor Complex–Fyn–NMDAR/PSD-95 and Fyn–tau axes, while preserving the physiological synaptic plasticity function of Fyn.

Based on these understandings, this review positions Fyn at the intersection of multiple pathways in tauopathy and related neurodegenerative diseases. We focus on the key roles of Fyn in the regulation of synaptic localization of NMDARs, Fyn–tau interaction, amplification of postsynaptic toxic signals, amplification of glial-related inflammation, shaping of the pathological propagation environment, as well as targeted intervention strategies and translational bottlenecks. We aim to provide a clearer framework for understanding the molecular coupling among tau pathology, synaptic imbalance, proteostatic stress, and neuroinflammation, thereby providing a foundation for the design of next-generation “mechanism-guided” therapies.

## Physiological functions of Fyn in the central nervous system (CNS)

### Structure and biochemical features of Fyn kinase

**Fig. 1:**
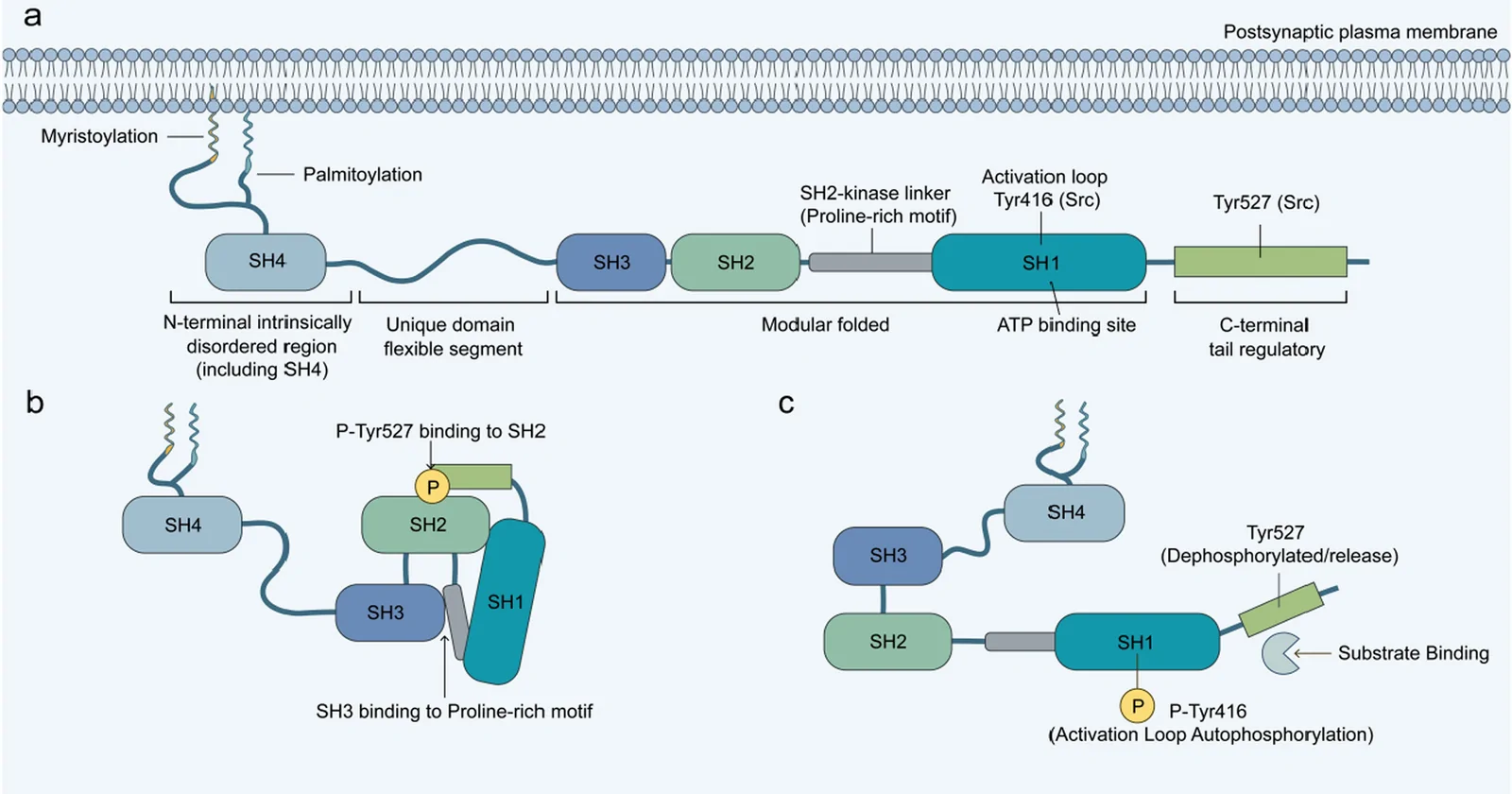
Structural framework and activation mechanism of Fyn kinase. **a** Fyn possesses a typical “modular” structural framework comprising an N-terminal membrane anchoring region (SH4 domain) carrying myristoylation and palmitoylation modifications, a Unique domain, SH3 and SH2 domains, an SH2-kinase linker, a catalytic protein tyrosine kinase domain (SH1), and a C-terminal tail regulatory region. The N-terminal region (SH4 and Unique domains) exhibits intrinsically disordered characteristics. This architecture enables Fyn to achieve fine coupling across “substrate/adapter protein recruitment–conformational switching–catalytic output”. **b** In the resting state, Fyn is stabilized in an auto-inhibited conformation: the phosphorylated C-terminal inhibitory site (Tyr527, Src numbering) binds to the SH2 domain, while the SH3 domain interacts with a proline-rich motif in the linker region. **c** Upon activation, dephosphorylation of the C-terminal site unlocks the kinase domain conformation and induces autophosphorylation of the activation loop site (Tyr416, Src numbering), which significantly enhances signal output

Fyn is a core member of the Src family kinases (SFKs) and has a typical molecular structural architecture. It encompasses an N-terminal membrane-anchoring segment (SH4, featuring myristoylation and palmitoylation modifications), a Unique domain, SH3 and SH2 domains, an SH2-kinase linker, a kinase catalytic domain SH1, and a C-terminal tail regulatory region housing crucial regulatory sites (Fig. 1a). The N-terminal region (SH4 and Unique domains) possesses intrinsically disordered characteristics [39–41]. This structure enables Fyn to achieve fine coupling of substrate/adapter protein recruitment, conformational switching, and catalytic output. At resting state, the phosphorylated C-terminal inhibitory site (Tyr527, Src numbering) binds to the SH2 domain, while the SH3 domain interacts with a proline-rich motif in the linker region, stabilizing an auto-inhibited conformation (Fig. 1b). Dephosphorylation of the C-terminal site unlocks the conformation of the kinase domain and induces autophosphorylation of the activation loop site (Tyr416, Src numbering), significantly enhancing the signal output (Fig. 1c**)** [42–44]. Lipid modification at the N-terminus confers preference to membrane microdomain, facilitating synaptic plasticity and phosphorylation of receptors. In tauopathies, tau guides Fyn to the dendritic or postsynaptic regions, where Fyn phosphorylates NMDARs and links them to PSD-95, forming a key molecular hub that amplifies synaptic toxicity [3].

### Genomic and genome-wide association study (GWAS)-based insights into Fyn kinase

Beyond the well-established cellular pathways, Fyn kinase induces neuroinflammation and excessive hyperphosphorylation of the tau protein. Large-scale GWAS have established an association between *FYN* polymorphisms and an elevated susceptibility to neurodegenerative diseases. A recent whole-genome sequencing study in a Chinese population revealed that the risk variant *FYN* rs997368 serves as a significant genetic risk factor for Parkinson’s disease (PD) [45]. In the context of AD, targeted genetic analysis further validated that *FYN* polymorphisms are closely associated with established pathological biomarkers. The intronic variant rs7768046 is correlated with elevated levels of phosphorylated tau and total tau in cerebrospinal fluid. The 3ʹ untranslated region variant rs1621289 is associated with increased level of Fyn protein in the hippocampus of AD patients [46, 47]. Data suggest that *FYN* variants are not only implicated in post-transcriptional regulatory mechanisms but also modulate the downstream tau pathway.

The impact of *FYN* polymorphism on the risk of sporadic AD may vary across ethnic groups [48]. Studies combining genetic and neuroimaging approaches still support the pathological role of Fyn. A recent imaging genetics study using tau positron emission tomography (tau-PET) found that the *FYN* rs57650567 was significantly associated with abnormal cortical tau deposition [49], and that it cooperates with the *APOE* ε4 allele to further increase tau burdens in the brain [49].

Fyn dysregulation also occurs at the transcriptome level through alternative splicing. The alternatively spliced, immune cell-predominant Fyn (FynT) is selectively increased in the neocortex of patients with AD and Lewy body dementias. This increase is further correlated with microglial activation, NFT progression, and cognitive decline. In contrast, the neuronal brain-predominant Fyn (FynB) remains relatively stable [50, 51]. Together, these genetic and transcriptomic findings, from GWAS-identified risk variants to isoform-specific splicing changes, further support Fyn as a key mechanistic regulator in a range of neurodegenerative proteinopathies.

### Fyn in synaptic development and plasticity

During nervous system development and maturity, Fyn, as the key tyrosine kinase node in the postsynaptic signal network, finely regulates the assembly and functional coupling of the postsynaptic glutamatergic receptor signaling complex. For example, PSD-95 acts as a molecular scaffold that anchors Fyn to the NMDAR. This interaction promotes the Fyn-mediated phosphorylation of NMDAR, thereby enhancing postsynaptic signal transmission and supporting activity-dependent synaptic plasticity processes [52]. Combined genetic and electrophysiological studies revealed that the Fyn signaling cascade plays a pivotal role in mediating synaptic plasticity, particularly LTP. Although Fyn deficiency does not affect basal synaptic transmission, paired-pulse facilitation, or post-tetanic potentiation, it results in blunting of LTP, triggering spatial learning and memory deficits [37, 38]. These findings indicate that Fyn is an important regulatory factor for maintaining synaptic plasticity and neural circuit function in physiological states [28, 44, 53].

### Fyn as a master regulator of oligodendrocyte (OL) myelination

In addition to its role in synaptic plasticity, Fyn has been implicated in myelination throughout the CNS. The peak of Fyn activity in the CNS matches the peak of myelination in the developing brain, and Fyn-deficient mice exhibit reduced myelination [54, 55]. Fyn is important for OL myelin formation, which was often neglected in past AD research. Fyn is a key driver of OL differentiation and myelin wrapping of axons. L1-CAM (L1 cell adhesion molecule) on the axonal surface binds to F3/Contactin on the OL membrane, thereby activating the intracellular Fyn kinase [56, 57]. The activated Fyn subsequently recruits and associates with cytoskeleton microtubule regulatory proteins (such as tau and Tubulin), driving the extension of oligodendrocyte processes and the myelin membrane wrapping [58]. Fyn plays a significant role in the synthesis of myelin basic protein, the principal protein within the myelin sheath, and is essential for the formation and maintenance of myelin sheath [54]. Fyn deletion leads to severe hypomyelination [59]. During normal development, oligodendrocyte precursor cells (OPCs) proliferate and migrate to their final destinations, after which they undergo terminal differentiation, extend cellular processes, and mature into functional myelinating oligodendrocytes [60]. Fyn contributes to the migration of OPCs by mediating the activation of cyclin-dependent kinase 5 (CDK5) through platelet-derived growth factor and by inducing the rearrangement of the actin cytoskeleton, which is conducive to migration [61]. Intracellularly, Fyn associates with integrins, leading to their phosphorylation and the subsequent activation of Rho family GTPases. This signaling cascade promotes actin dynamics, facilitating membrane ruffling and the formation of lamellipodia, enabling OLs to establish contacts with axons [62, 63]. Inhibition of Fyn activity has been demonstrated to impede the morphological differentiation of OPCs into mature OLs [62, 64]. In the early stages of AD and amyotrophic lateral sclerosis (ALS), the loss of white matter (mainly containing axons) integrity often precedes gray matter (mainly containing dendrites and cell bodies) atrophy [65, 66]. This finding supports the hypothesis that the degradation or disappearance of myelin precedes the occurrence of brain atrophy.

Fyn involves a complex “double-edged sword” pathological mechanism in neurodegenerative diseases. Excessive activation of Fyn is toxic to neurons through excessive activation and cumulative damage of intracellular signaling pathways, while impaired functions of Fyn lead to OL demyelination, resulting in the dysfunction of white matter network [65, 67]. These findings indicate that nerve degeneration is not an isolated pathological process affecting one cell type or one pathway, but a dynamic result from multiple pathological mechanisms. Strategies to maintain the balance between Fyn activation and inhibition may protect synapses and neural networks [68].

### Regulation of neurotransmitter receptors by Fyn

In the PSD, Fyn is a key non-receptor tyrosine kinase. It regulates the membrane localization and gating of several neurotransmitter receptors, and the timing of downstream signals mainly through two ways [3, 26, 69]. The first is by directly phosphorylating receptor subunits, and the second is through the NMDAR-associated protein complex, which locates and stabilizes the receptor and determines the intensity and duration of synaptic excitation transmission [28, 69]. Taking glutamatergic receptors as an example. Fyn is an important regulator of NMDAR signaling [28]. When its lipid modification (palmitoylation) is enhanced, it will be enriched in dendritic spine membrane microdomains, accompanied by increased phosphorylation levels of GluN2B. This promotes the open conformation of the receptor and amplifies Ca^2+^ influx, thereby increasing neuronal susceptibility to excitotoxic damage [70]. Therefore, GluN2B-selective antagonists can partially reverse this phenotype, indicating that the “Fyn–GluN2B” axis is a target for intervention [69, 71]. Under pathological conditions, upstream abnormal stimuli can lead to the imbalances association between Fyn and receptors. As previously discussed, Aβo binds to PrP^C^ and co-clusters with mGluR5, triggering Fyn activation and driving the synaptic toxicity cascade [30]. In addition, receptor-level signal amplification synergizes with calcium-dependent synaptic plasticity. Calcium/calmodulin-dependent protein kinase II alpha (CaMKII) plays a central role in synaptic plasticity and the AMPAR (α-amino-3-hydroxy-5-methyl-4-isoxazolepropionic acid receptor)-mediated neurotransmission [30, 72]. Concurrently, molecules associated with the neural inhibitory loop, including gamma-aminobutyric acid type A (GABA_A_) receptor subunits and glutamate decarboxylase 1/2, also exhibit adaptive regulatory changes [73, 74]. At presynaptic terminals, the modulation of voltage-gated Ca^2+^ channels and intracellular Ca^2+^ stores plays a critical role in regulating the release properties of neurotransmitters, including both glutamate and GABA [74, 75]. Elevated Ca^2+^ influx activates CaMKII, which functions at both presynaptic and postsynaptic sites to augment synaptic efficacy. At the presynaptic terminal, Ca^2+^-dependent activation of CaMKII promotes vesicle clustering and elevates the release probability of glutamate; at the postsynaptic site, CaMKII significantly potentiates AMPAR-mediated neurotransmission [72, 76]. When glutamate release becomes excessive, it overstimulates glutamate receptors and raises intracellular Ca^2+^ levels, initiating a toxic cascade that finally causes neuronal damage. This mechanism of glutamate-induced calcium overload is consistent with the general model of excitotoxicity, which is considered a common pathogenic pathway in various neurodegenerative diseases [77, 78].

### Fyn in cytoskeletal dynamics and axonal transport

In addition to participating in the regulation of receptor signaling, the Fyn-related pathways are also related to the stability of neuronal structures and substance transport. Fyn modulates neuronal migration through regulation of the cytoskeletal dynamics and the multipolar-bipolar transition of newly generated neurons during cortical development [79]. Semaphorin-3A (Sema3A) and Fyn are involved in cortical dendrite projection. Sema3A enhances CDK5 activity via phosphorylation at Tyr15, a phosphorylation site associated with Fyn [80]. Fyn and CDK5 mediate the axonal transport induced by Sema3A [81]. Furthermore, Dock3 induces axonal outgrowth by forming a protein complex with Fyn and the Wiskott-Aldrich syndrome protein (WASP) family verprolin-homologous protein (WAVE) proteins at the plasma membrane, downstream of the brain-derived neurotrophic factor (BDNF) signaling [82]. Interactions between Fyn and tau, which mediate microtubule assembly, also promote the transport of cargoes, such as myelin, towards the axon and the synthesis of myelin itself [56, 83].

By employing the CRISPR/Cas9 system to substitute adenine with thymine at position 999 in the endogenous *ZDHHC21* gene, Li et al. generated a ZDHHC21^T209S/T209S^ knock-in mouse model that exhibits impaired spatial learning, Aβ accumulation, tau phosphorylation, and aberrant Fyn palmitoylation-associated synaptic impairments (reduced dendritic branching and spine density), all of which could be partially rescued via palmitoylation inhibition [84]. Building upon these findings, extensive literature suggests that aberrant Fyn activation may indirectly disturb the neuronal polarity maintenance and long-range transport efficiency by affecting the structural plasticity-related cytoskeletal network (e.g., via the Fyn-tau-microtubule axis). Studies in multiple transgenic and cellular models have shown that Fyn interacts with tau and promotes neurotoxicity and synaptic dysfunction in a tau-dependent manner [29]. Pathological changes in tau protein cause damage to axonal transport. For example, tau hyperphosphorylation at the AT8 site increases microtubule spacing. Such structural changes disrupt the anterograde transport of organelles including mitochondria [85, 86]. Collectively, these findings provide a crucial mechanistic framework for understanding impaired microtubule stability and axonal transport deficits in disease states.

## Upstream regulatory mechanisms controlling Fyn activation and localization

The function of Fyn is determined not only by its expression level, but also by transcriptional and translational regulation, post-translational modification, and dynamic changes in subcellular location [47, 67, 87]. Under physiological conditions, these regulatory mechanisms ensure the reversibility and localization of Fyn signals. Under pathological states, the imbalance of the upstream regulatory network drives pathological continuous activation [67, 88, 89]. Therefore, understanding the key mechanisms that regulate the activity and positioning of Fyn is critical for understanding the transformation of its role in tauopathies.

### Transcriptional and post-transcriptional regulation of Fyn expression

The expression of Fyn varies significantly across different brain regions, developmental stages, and cell types. Moreover, its transcriptional regulation is affected by a variety of transcription factors and epigenetic mechanisms [47, 67]. The microRNA-mediated post-transcriptional regulation finely regulates the level of Fyn protein, thus limiting the intensity and duration of its signal output [90]. Under physiological conditions, these mechanisms are interrelated to maintain moderate expression of Fyn and controllable signal conduction. In the pathological context, this equilibrium system is disturbed, which may cause abnormal accumulation of Fyn and trigger continuous signal amplification, thus promoting downstream pathological processes [89, 90].

### Post-translational modifications governing Fyn activity

Post-translational modifications, especially phosphorylation of key tyrosine residues, modulate Fyn activity [67, 91]. As described earlier, the phosphorylation states of the C-terminal inhibitory site and the activation-loop site govern the equilibrium between the closed, autoinhibited and the open, active conformations of Fyn [42–44]. In addition to phosphorylation, ubiquitination limits signal accumulation by regulating protein stability and degradation rates. Meanwhile, lipid modifications (e.g., N-terminal fat acylation-related modification) promote the directional recruitment of Fyn to membrane compartments/lipid rafts and reshape the substrate accessibility. Under conditions such as inflammation and oxidative stress, redox-related modifications can further change the interaction spectrum and subcellular distribution of Fyn [92–94]. These regulatory mechanisms enable rapid and reversible response of Fyn to external stimuli.

### Subcellular localization and compartment-specific activation of Fyn

The function of Fyn is related to its subcellular localization. The distribution of Fyn within the PSD, membrane lipid rafts, and intracellular vesicle system determines the substrates it contacts [87, 95]. Within excitatory synapses, scaffold complexes, including PSD-95/SAPAP/SHANK, form an organized signaling platform. This platform establishes a connection between the receptor complex and the cytoskeleton as well as the molecular motor system, thereby confining Fyn signaling spatially and endowing it with high specificity [96]. However, in the context of diseases, this localization control may be broken. The restriction imposed by membrane microdomains and scaffolds is lost, and Fyn may undergo abnormal aggregation in synaptic regions or compartments associated with endosomal vesicle. This abnormal redistribution may lead to overactivation of Fyn, and amplify pathological signal transduction and neurotoxic cascades [3, 87].

### Extracellular cues and signaling pathways modulating Fyn

Many extracellular signals, such as neurotrophic factors, inflammatory and chemotactic factors, and cell–cell interaction signals, can activate the SFK pathway through receptors and adapter or scaffold protein networks. Through spatially and temporally well-organized recruitment, these signals can finely control the activation threshold of Fyn and the intensity of its signaling output [28, 87]. In neurodegenerative diseases, soluble Aβ can directly or indirectly interact with several membrane receptors, including NMDARs, mGluR5, α7-nicotinic acetylcholine receptor, p75 neurotrophin receptor, receptor for advanced glycation endproducts, and erythropoietin-producing hepatocellular carcinoma receptors (Eph receptors). These interactions provide continuous external input for downstream kinase signaling in the synaptic region [97, 98]. Meanwhile, glial cells under chronic activation can continuously release inflammatory mediators such as tumor necrosis factor-α (TNF-α), interleukin (IL)-6, and IL-1α. This leads to local inflammatory amplification and can turn signaling events, which are normally transient and reversible, into a persistent state. As a result, the Fyn pathway is more prone to dysregulation, and pathological signals are more inclined to accumulate [99–101]. Studies have indicated that Fyn participates in BDNF/Tropomyosin receptor kinase B (TrkB) signaling [102], neurotransmitter receptor regulation [28, 69], and lipopolysaccharide (LPS)/TLR4 (Toll-like receptor 4)-mediated inflammatory signaling in microglia [100], acting as a key hub linking external stimuli to internal responses [102].

## Fyn–tau interactions and tau-mediated synaptic toxicity

Studies have demonstrated important interactions between Fyn and tau. Fyn affects tau phosphorylation, subcellular distribution, and aberrant synaptic accumulation, enhancing the toxicity of tau at synapses. This in turn amplifies Fyn signaling [3, 19, 103, 104]. Unraveling the molecular basis of the Fyn–tau axis could help explain how tau pathology progresses to cause synaptic and functional dysfunction.

### The biophysics of toxicity: liquid–liquid phase separation (LLPS)-mediated Fyn recruitment

LLPS refers to a biological or physicochemical process that drives the formation of granular or droplet-like condensates characterized by a high concentration of macromolecules within the compartments. These structures with high protein density are referred to as complex coacervates [105, 106]. A key characteristic of the aggregates is that they exhibit an extremely high protein concentration within a minute volume compared to the solute phase; furthermore, the proteins within these compartments are all in a hydrated state and capable of free exchange [107].

Tau protein, particularly the proline-rich region, is an intrinsically disordered protein [108]. Under pathological conditions, it has a high propensity for LLPS, giving rise to high-concentration biomolecular condensates [109]. Recombinant tau protein that is phosphorylated or mutated and exhibits aggregation-prone properties undergoes LLPS [109]. Both physiological and pathological tau phosphorylation, as well as disease-associated mutations, promotes LLPS of tau [110–112].

The interaction between Fyn and tau is not a simple “lock-and-key” combination, but involves a complex biophysical phase transition process. Fyn-SH3 engages tau through multivalent interactions across multiple hot spots, and tau condensates may facilitate local Fyn enrichment and trapping [103, 113]. The high local concentration of PXXP motifs in tau condensates greatly enhances the binding kinetics with Fyn-SH3. This phase separation environment gives rise to a microreactor effect, which concentrates the Fyn kinase activity in specific subcellular regions. The kinase activity of Fyn is regulated via its conformational transition between two states: an inactive, closed conformation that lacks the capacity for substrate binding, and an active, open conformation that enables substrate interaction and the execution of catalytic function. The conformational transition of Fyn from a closed state to an open state promotes its molecular clustering [114]. Alterations within dendritic spines also lead to a shift in the conformational equilibrium of Fyn, favoring the “open” (activated) conformation [113]. Pathogenic tau mutations related to frontotemporal lobar degeneration (FTLD), such as P301L, increase the stability of abnormal aggregates by enhancing the hydrophobic interactions/dehydration of tau, resulting in the aberrant immobilization and trapping of Fyn in postsynaptic dendrites and overactivation of toxic signals [20, 113, 115, 116]. A possible mechanism is that the LLPS of the tau-P301L mutant facilitates the formation of membraneless biomolecular condensates, which function as subcellular hubs for enrichment of Fyn molecules, maintaining them in an immobilized, ordered, and compartmentalized state. This in turn promotes their interaction with downstream signaling proteins [113]. Fyn has also been shown to assemble into nanodomains, which are also known as nanoclusters. These structures, measuring nanometers in scale, act as functional platforms that facilitate the concentration of diverse molecules and the integration of signaling cascades [117, 118].

### Kinase-mediated tau mislocalization and Fyn coupling

Calcium-dependent kinase networks drive the abnormal phosphorylation of tau, causing tau to mislocalize to dendrites where it abnormally recruits Fyn to postsynaptic receptors [2, 6, 23, 119]. Pathological stimuli (e.g., neuronal hyperexcitability induced by Aβo) are capable of activating the CAMKK2 (calcium/calmodulin-dependent protein kinase kinase 2)–AMPK (AMP-activated protein kinase) pathway. This activation leads to the preferential phosphorylation of the KXGS sequence (typified by Ser262) of tau within the microtubule-binding region, thereby diminishing its affinity for microtubules. Moreover, it facilitates the translocation of tau from axons to dendrites and dendritic spines. This mislocalized dendritic tau further acts as a molecular scaffold, promoting coupling of the SFK Fyn with NMDAR complexes and stabilizing receptor anchoring in the PSD. This amplifies downstream excitotoxic signals and accelerates synaptic structural and functional damage. Consequently, this kinase-mediated phosphorylation and mislocalization of tau, followed by the coupling with Fyn, can be regarded as a crucial juncture at which tau transforms from a microtubule-stabilizing factor to a synaptic toxic effector. Blocking the Fyn–tau interaction or targeting its upstream kinase pathways may offer synaptic protection [119].

### Fyn–tau complexes at the PSD

In tauopathies such as AD, the accumulation of abnormal Fyn–tau complexes in the PSD is an important mechanism linking tau mislocalization to synaptic damage [120–122]. Mislocalized tau protein acts as a scaffold to recruit Fyn into the PSD-95–NMDAR signaling complex [23, 123]. This abnormal coupling enhances the NMDAR-associated excitatory signaling and Ca^2+^-dependent toxic cascades, contributing to impaired synaptic plasticity and disrupted receptor homeostasis [3, 23, 29, 52, 124]. Therefore, the mislocalized Fyn–tau-NMDAR axis reduces the resistance of synapses to pathological stimuli, including the upstream Aβ-related signals. This mechanism can also explain that the Fyn-driven NMDAR hyperactivation in specific regions underlies the selective early synaptic toxicity of tau pathology.

### Tau toxicity amplified by Fyn signalling

**Fig. 2:**
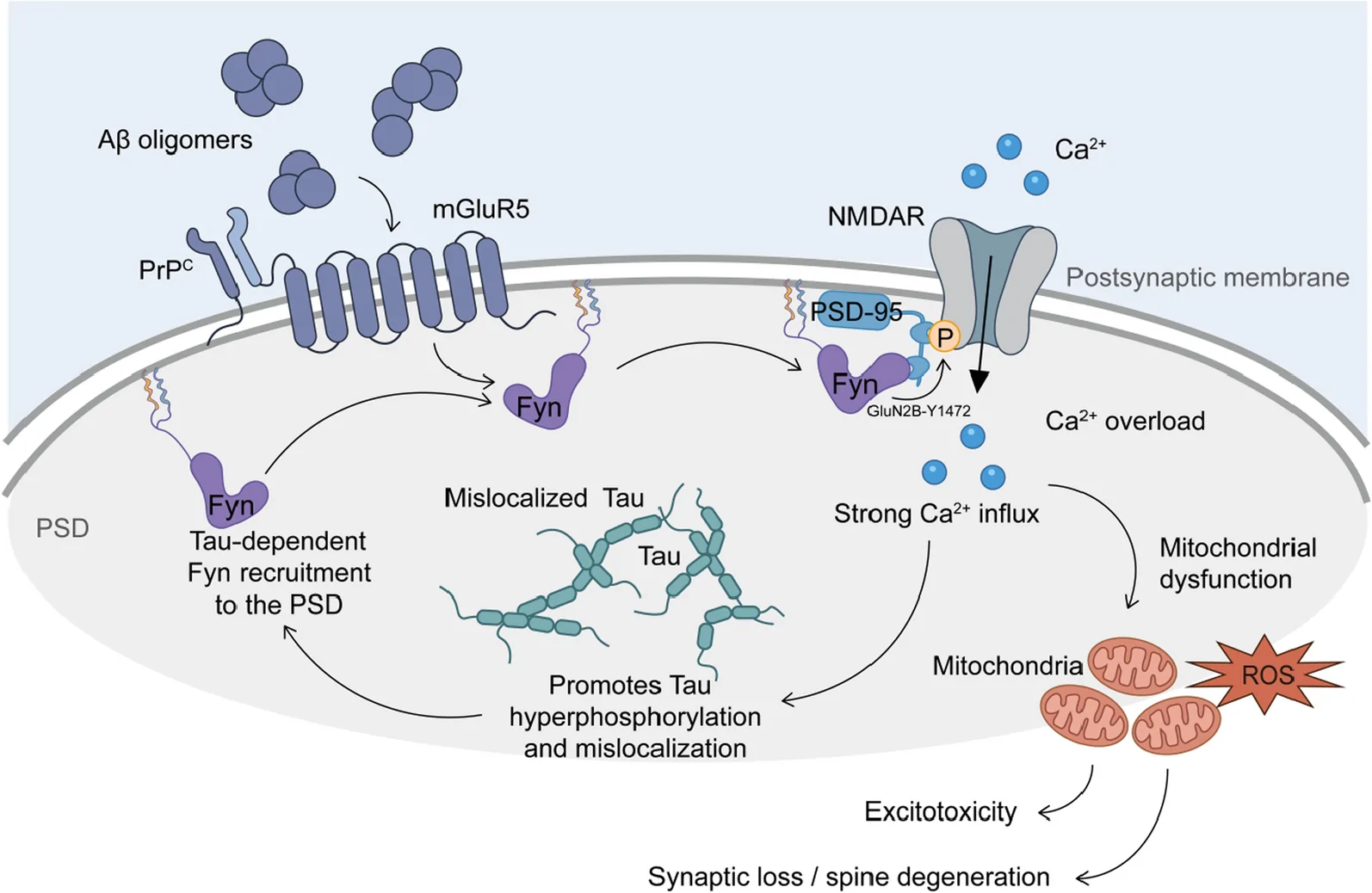
Fyn kinase acts as a critical hub integrating Aβ- and tau-mediated synaptic toxicity at the PSD. Aβo initiates the pathogenic process through binding to the PrP^C^–mGluR5 complex on the postsynaptic membrane, subsequently activating Fyn kinase. The initial Ca^2+^ influx promotes tau hyperphosphorylation and prompts the translocation of tau into dendritic spines. The mislocalized tau subsequently recruits Fyn to the PSD. In the PSD, Fyn interacts with the scaffold protein PSD-95 and phosphorylates the GluN2B subunit of NMDAR at Tyr1472. This phosphorylation strengthens the interaction between NMDAR and PSD-95 and causes a large and sustained Ca^2+^ influx. The excessive Ca^2+^ influx further promotes tau hyperphosphorylation and exacerbates tau accumulation in the PSD. As a result, tau continues to recruit Fyn, and NMDARs remain overactivated, which further worsens intracellular Ca^2+^ overload. The persistent Ca^2+^ overload also causes severe mitochondrial dysfunction and increases ROS accumulation, finally leading to excitotoxicity and progressive synaptic loss or spine degeneration

In tauopathies, Fyn is a downstream facilitator of tau toxicity [125]. Abnormal activation forms a self-sustaining positive feedback loop driven by excitotoxicity and calcium imbalance, making tau-related neuronal injury more severe [3, 126–129]. In AD, which is considered a secondary tauopathy, Aβo disrupts the Fyn/NMDAR signaling pathway through the PrP^C^–mGluR5 axis [30, 122, 129]. This process increases cytoplasmic Ca^2+^ levels [125], which then promotes excessive tau phosphorylation and drives tau mislocalization to dendrites and dendritic spines [1, 6, 119, 130–133]. Tau mislocalization recruits Fyn to the PSD [125, 134], where Fyn phosphorylates GluN2B at Y1472, augmenting the NMDAR-dependent synaptic Ca^2+^ currents, subsequently resulting in synaptic dysfunction and structural damage [2, 3, 30, 125, 135, 136]. Simultaneously, persistent Ca^2+^ overload impairs mitochondria [126, 129]. It can cause mitochondrial Ca^2+^ overload, impair oxidative phosphorylation, and increase reactive oxygen species (ROS) production [129]. In turn, mitochondrial dysfunction and ROS reduce the cell’s capacity to buffer Ca^2+^ and further disrupt calcium homeostasis through influencing the activity of intracellular ER calcium channel **(**Fig. 2**)** [129]. In this way, a detrimental effect manifests along the “Fyn–NMDAR–Ca^2+^–mitochondria/ROS” axis [126, 129]. This expedites the transition from early tau-associated synaptic impairment to more extensive neurodegeneration [137–139].

## SFK-driven synaptic dysfunction and neuronal network failure

Synaptic dysfunction represents an early and crucial pathological event in neurodegenerative diseases [16, 140]. SFKs, including Fyn and Src, serve as PSD signaling molecules that modulate the tyrosine phosphorylation of NMDARs and the propagation of excitatory signals [52, 70]. Under physiological conditions, these kinases are involved in learning, memory, and synaptic plasticity [52, 70]. Nevertheless, when the overall activity of SFKs is abnormally up-regulated, often triggered by Aβo and propagated by tau, extensive excitotoxicity may occur, causing destruction of the cytoskeletal structure and neuroinflammation. With accumulation of synaptic damage, neuronal stress responses and cell death pathways are gradually activated, inducing a profound functional imbalance at the neural network level and driving disease progression towards terminal stages of neurodegeneration [29, 141].

### Src family kinases

The SFK family encompasses nine members, namely Src, Fyn, Lck, Hck, Yes, Lyn, Blk, Fgr, and Yrk. Stimulation of SFKs exhibits a positive correlation with microglial activation and neuropathological states. The homeostatic functions of microglia, including phagocytosis, environmental sensing, and secretion of inflammatory mediators, are regulated by SFKs [142]. Beyond microglial activation, SFKs are also associated with microglial migration or chemotaxis [143].

Src and Fyn are the principal members of SFKs expressed in neurons and glial cells. In neurons, Fyn and Src are associated with excitatory and inhibitory ion channels and can modulate both NMDA and GABA_A_ receptors [143]. In contrast to c-Src, Fyn may function as a negative regulator of microglial phagocytosis [144]. Exposure to Aβo induces Fyn activation in neurons and c-Src activation in microglia [35, 145]. This combination results in synapse loss and behavioral impairments associated with AD.

Fyn mediates interactions among Aβ, tau, NMDARs, and neuroinflammation, serving as a “universal target” for multiple diseases [88]. Although both Src and Fyn are involved in Aβ toxicity, the neuroprotective effect of Fyn knockdown was significantly stronger than that of Src knockdown, indicating the dominant role of Fyn in mediating Aβ neurotoxicity, which cannot be compensated by Src [146].

Of the five Src-family non-receptor tyrosine kinases expressed in the CNS (Lck, Lyn, Fyn, Src, and Yes), Src and Fyn have been confirmed as part of the PSD [28, 147–150]. In this network, PSD-95 serves as an important scaffold protein and connects key signaling molecules. Fyn binds to PSD-95 through interactions of its SH2 domain with the third PDZ domain of PSD-95 [52, 151]. This binding keeps Fyn close to the NMDAR complex, supporting the Fyn-mediated tyrosine phosphorylation of NMDAR. This finding also suggests a wider possibility that other SFK members may also enter synaptic signaling networks through similar interactions with PSD-95 [151].

### Synergistic regulation of NMDAR signaling by SFKs

In the PSD, SFKs enhance the function of NMDARs, which is a key mechanism for regulating NMDAR-dependent synaptic potentiation [28]. Under physiological conditions, the endogenous tyrosine kinase Src directly regulates the activity of NMDARs, increasing the channel open probability [152]. PSD-95 binds to and clusters with NMDARs and promotes the Fyn-mediated tyrosine phosphorylation of the GluN2A subunit by facilitating formation of the GluN2A–PSD-95–Fyn ternary complex [22, 52]. Although there are shared interactions, Src and Fyn have different phosphorylation targets: Src predominantly mediates the phosphorylation of the GluN2A C-tail (e.g., Y1325), while Fyn mainly mediates the phosphorylation of the GluN2B C-tail (e.g., Y1472) [22, 28].

### Synaptic excitotoxicity and aberrant SFK–tau interactions

SFKs have direct interactions with tau, which is regarded as a key molecular link in the “tau synaptic mislocalization–signal disruption” axis of tauopathies. Tau is an axonal protein. Its mislocalization in dendrites would bring Fyn to the postsynaptic site [3]. This has a clear structural basis. The proline-rich middle region of tau has a PXXP motif, which can bind to the SH3 domain of SFKs. Recent structural studies show that this binding does not rely on a single critical motif but rather follows a pattern of “multi-hotspot adaptive binding”. In this pattern, the SH3 domain and several hotspot regions of about 85 residues interact with full-length tau, and most of the interactions are moderate or weak [103]. Since tau has multiple binding sites, even if one consensus motif is deleted, it can still act as a flexible recognition surface [103].

This adaptable binding mechanism is particularly detrimental during disease progression. In AD, tau is hyperphosphorylated and aberrantly redistributed into the somatodendritic compartment [3]. Although widespread pathological phosphorylation may change the overall binding state, tau still retains a strong ability to anchor Fyn at the PSD because it has several alternative binding sites. This means that tau can continue to recruit Fyn even when some PXXP motifs lose function [103]. In addition, these binding sites are separated by long and highly flexible linker regions. This structural feature allows tau to bind several SH3-containing proteins at the same time. As a result, tau can recruit not only Fyn, but also other SFK members such as Src and Lck [18, 103]. In the end, the abnormal somatodendritic accumulation of tau and its persistent docking capacity lead to excessive recruitment of SFKs. This process directly links NMDARs to downstream excitotoxic signaling and ultimately causes severe synaptic dysfunction [3].

### SFK-mediated cytoskeletal instability and transport failure

In AD models, the interaction between tau and Fyn plays a key role in Aβ-induced synaptic toxicity and neuronal death [134]. This abnormal SFK-tau signaling also disrupts normal cytoskeletal function. Similar as genetic mutations that reduce tau binding to microtubules and promote formation of hyperphosphorylated filamentous aggregates [153], kinase-mediated modifications can also cause tau detachment from the cytoskeleton. The detached tau is more likely to undergo hyperphosphorylation and proteolytic cleavage, which promotes the formation of NFTs [154].

Tau detachment also results in cytoskeletal instability, which seriously disrupts intracellular transport. This disruption directly contributes to the development of adult-onset neurodegenerative diseases, which are a group of age-related disorders marked by progressive neuronal dysfunction and selective neuronal loss [155]. Neurons rely on efficient, microtubule-based transport to deliver essential cargoes to distal nerve terminals. Damage to both structure and transport makes the synapse a major site of vulnerability during neurodegeneration [156]. In fact, many past treatments may have failed to halt the clinical progression of neurodegenerative diseases because they did not provide enough protection for these critical synaptic regions [156].

### From synaptic failure to neuronal loss: intrinsic damage and neuroinflammation

**Fig. 3:**
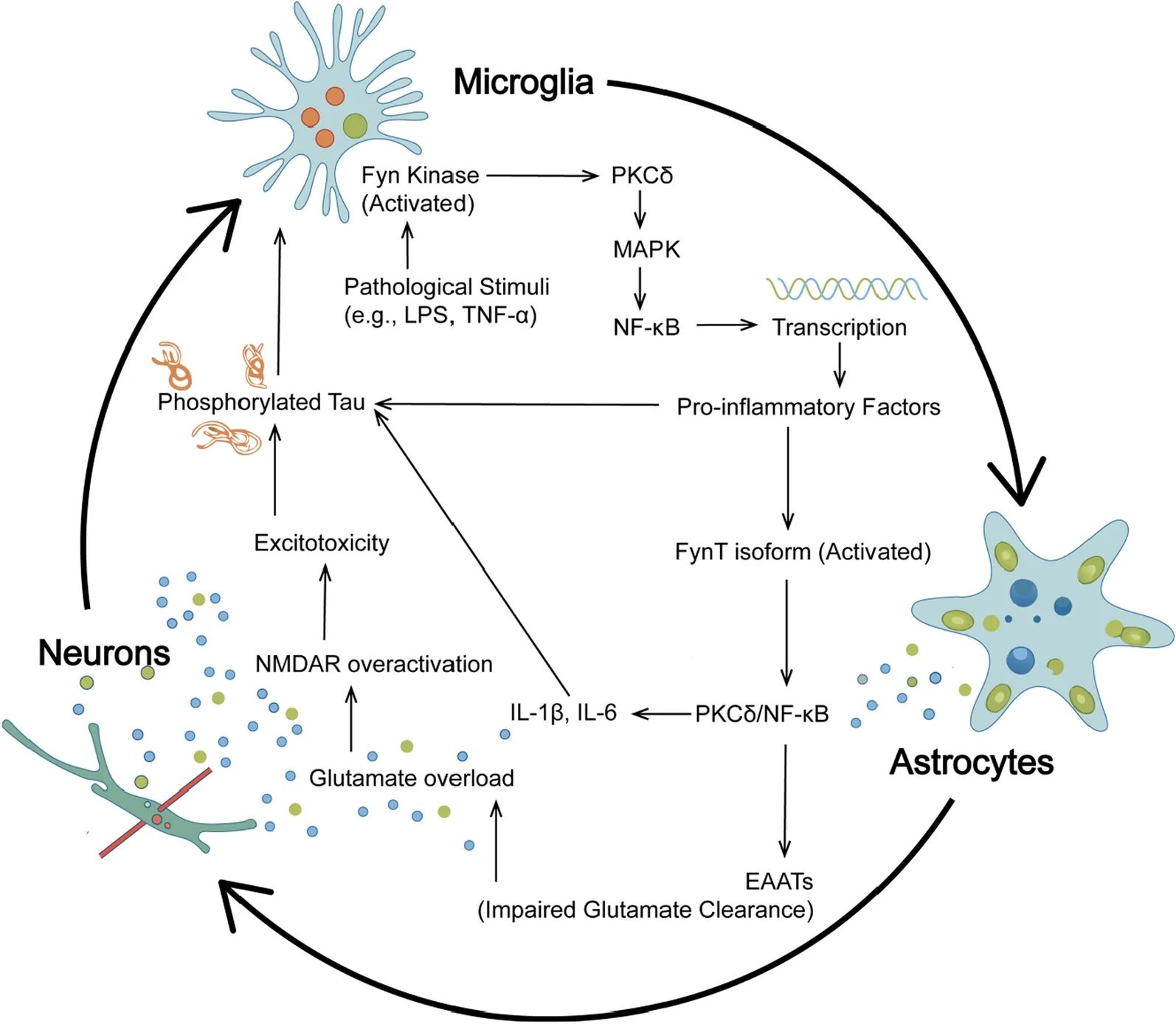
The Fyn kinase-mediated vicious feed-forward cycle of neuroinflammation, excitotoxicity, and tau pathology. Pathological stimuli (e.g., LPS or TNF-α) initially activate Fyn kinase in microglia, triggering the PKCδ–MAPK–NF-κB signaling cascade, driving the transcription and substantial release of pro-inflammatory cytokines. These inflammatory mediators within the microenvironment subsequently exert an effect on astrocytes, selectively upregulating and activating the FynT isoform. Aberrant FynT activity within astrocytes not only exacerbates the secretion of pro-inflammatory cytokines (e.g., IL-1β and IL-6) via the PKCδ/NF-κB pathway, but also reduces the protein levels of GLT-1, a component of the EAATs family, resulting in defective glutamate clearance from the synaptic cleft. The resultant glutamate overload causes pathological over-activation of neuronal NMDARs, inducing synaptic excitotoxicity. Ultimately, pro-inflammatory cytokines such as IL-1β and IL-6 promote aberrant tau phosphorylation in neurons. The released pathological phosphorylated tau in turn serves as a stimulant to further activate microglia. At last, a self-sustaining and amplifying “inflammation–tau” vicious cycle among microglia, astrocytes, and neurons is established

Many chronic neurodegenerative diseases typically commence with localized synaptic impairment, which subsequently progresses towards the irreversible loss of specific neuronal populations [157]. Fyn plays a central role in this process. When Fyn engages in an interaction with Aβ and tau, it facilitates the Aβ-induced synaptic toxicity and the subsequent neuronal death [36, 134]. Excitotoxicity serves as a primary driving factor for this alteration. Under normal conditions, NMDARs are essential for synaptic plasticity and neural development. However, once these receptors are hyperactivated, they would cause excitotoxic damage **(**Fig. 3**)** [70]. This excessive activation leads to a rapid increase in extracellular Ca^2+^ influx [158]. The excessive Ca^2+^ subsequently penetrates into mitochondria, leading to mitochondrial calcium overload. This phenomenon represents a crucial event in glutamate-induced neurotoxicity [158]. Coupled with this oxidative stress, aberrant protein handling, including ER stress and activation of the UPR, further exacerbates the disruption of cellular energy balance. These alterations ultimately disrupt intracellular homeostasis and promote neurodegeneration [157].

Simultaneously, damaged synapses are also influenced by neuroinflammation, and microglial activation represents a prevalent pathological characteristic in the AD brain [159]. Aβo induces neurotoxic responses and prompts microglial transition towards a pro-inflammatory state [160]. This Aβ-induced microglial activation is closely linked to non-receptor tyrosine kinase signaling. In particular, several microglial populations in the brains of AD patients exhibit immunoreactivity for the active phosphorylated forms of Lyn and Src [159, 160]. In vitro studies also showed that Aβo increases the overall protein phosphotyrosine in primary microglia and the release of pro-inflammatory cytokines, including TNF-α and IL-6 [159, 160]. This process can be ameliorated by broad-spectrum inhibitors, such as the Src/Abl inhibitor dasatinib [160], as well as more selective small molecules like LDDN-0003499. Consequently, these compounds also decrease the Aβ-induced cytokine release [159].

Ultimately, the Fyn/NMDAR-mediated excitotoxicity within neurons and the Lyn/Src-driven microglial inflammation jointly exert a severe detrimental impact on neuronal survival. Suppression of these kinase pathways can yield distinct neuroprotective effects. For instance, deletion of Fyn can impede behavioral impairment and the loss of synaptic proteins in disease models associated with P301L tau [36]. These findings suggest that targeting both synaptic toxicity and microglial activation might serve as an effective strategy to slow or impede the progression from synaptic failure to end-stage neurodegeneration [134, 159].

### A broader comparison of Fyn with SFK family members

SFK members share a conserved modular architecture comprising an N-terminal SH4 domain, a unique region, SH3 and SH2 domains, an SH1 kinase domain, and a C-terminal regulatory tail [161–163]. Immediately downstream of the SH4 domain lies a “unique” region comprising 50 to 70 amino acid residues, which exhibits significant sequence divergence across different family members [161]. Therefore, the structural homology provides the basis for partial functional overlap but does not support complete interchangeability of function.

The intracellular trafficking of SFKs is determined by the palmitoylation state of their SH4 domains [164]. Yes represents a monopalmitoylated SFK, whose post-synthetic delivery proceeds from the Golgi apparatus to the plasma membrane. The palmitoylated Lyn is also transported to the plasma membrane via exocytosis through the Golgi region along the secretory pathway [165]. By contrast, Fyn with dual palmitoylation is directly sorted to the plasma membrane [164]. The nonpalmitoylated Src exhibits rapid exchange between the plasma membrane and late endosomes/lysosomes [166]. Collectively, the trafficking of SFKs is determined by their palmitoylation status. Due to the variations in subcellular localization among SFK members, the functional loss of Fyn cannot be fully compensated by other members.

Five members of the SFK family, namely Lck, Lyn, Fyn, Src and Yes, are expressed in the CNS [151]. Although Src and Fyn play important roles in synaptic transmission and plasticity at excitatory synapses [28, 147–151], the other three members have also been detected in the PSD [151]. Lyn, Yes and Src, but not Lck, exhibit physical interaction with PSD-95 [151]. PSD-95 plays an essential role in the modulation of NMDAR activity mediated by Fyn and other members of SFKs, functioning as a molecular scaffold that tethers these protein tyrosine kinases to the GluN2A subunit. Different from PSD-95, other PSD-95 family members including PSD-93, synapse-associated protein (SAP) 97, and SAP102 do not physically interact with Lyn, Src or Yes [52, 151]. These observations indicate that other SFK members can enter the synaptic complex but are not fully equivalent substitutes for Fyn.

Although both Lck and Fyn preferentially phosphorylate Tyr18, Lck exhibits moderately higher phosphorylation activity against other tyrosine residues compared with Fyn [167]. These findings suggest that Fyn and Lck share tau-directed kinase activity but have distinct substrate preferences, arguing against complete functional redundancy.

Members of the SFK display distinct non-redundant functions in microglia, and these functions vary across disease contexts [142]. c-Src is commonly associated with proinflammatory activation, generation of ROS, and specific phagocytic responses [142]. In contrast, Fyn functions as the primary upstream enhancer of the PKCδ (protein kinase Cδ)–MAPK (mitogen-activated protein kinase)–NF-κB (nuclear factor kappa B) signaling [100], α-synuclein (α-syn) internalization, mitochondrial ROS production, NOD-like receptor family pyrin domain-containing 3 (NLRP3) inflammasome activation [168], and Kv1.3-mediated inflammation in the context of PD [169]. In the context of PD models, Lyn mediates the phosphorylation of F-actin-associated protein cortactin after sensing the CD11b–Nox2-derived hydrogen peroxide (H_2_O_2_), which then facilitates directional microglial migration in response to α-syn [170]. Meanwhile, this kinase acts as a negative regulator of Syk-dependent disease-associated microglial transformation and Aβ phagocytosis [171]. Conversely, studies in two independent APP models suggest that Hck plays a role in promoting microglial Aβ phagocytosis, plaque wrapping and early inhibition of amyloid pathology [172]. Collectively, these findings indicate that microglial SFKs are not functionally interchangeable; rather, they engage distinct receptor–effector modules and may exert divergent effects on inflammation, migration, and amyloid clearance depending on the disease context and the pathological stage.

## Glial Fyn signaling and neuroinflammatory amplification

Fyn activity in microglia and astrocytes is also important in disease development. More studies show that Fyn signaling can regulate glial activation, the release of inflammatory factors, and glia-synapse interactions [100, 173]. In tauopathies, dysregulated Fyn signaling in glial cells may transform protein homeostasis imbalance into chronic neuroinflammation. This process may exacerbate neuronal damage [51, 100, 174]. Signaling between cell types drives disease progression over time.

### Fyn in microglial activation and inflammatory signaling

In microglia, Fyn participates in both the start and the amplification of inflammatory responses [100, 142]. Under normal conditions, microglia function to maintain brain homeostasis. They regulate synapses, support neurons, and remove apoptotic or damaged cells through phagocytosis and clearance [175, 176]. However, upon nervous system injury or chronic pathological stimulation, this functional balance is disrupted [175, 177]. Evidence shows that Fyn, Src, and other SFKs are not only associated with microglial proliferation, differentiation, and migration, but also exhibit an upregulation tendency in diverse nerve injury contexts. Hyperactivation of these kinases can result in the “amplification” of downstream inflammatory signals [142, 178]. For instance, in BV2 microglia, LPS and TNF-α stimulation can induce the upregulation of Fyn expression and activity [100]. A previous study in microglia from Fyn knock-out mice indicates that the activity of Fyn kinase is essential for the phosphorylation of PKCδ and the activation of the MAPK–NF-κB axis, which initiates the transcriptional program of pro-inflammatory genes [100]. During this process, NF-κB regulates the expression of pro-inflammatory factors like TNF-α and IL-1β **(**Fig. 3**)** [100]. Therefore, abnormal Fyn activity may amplify the response of microglia to pathological stimuli by increasing the release of pro-inflammatory mediators, inducing a chronic inflammatory microenvironment in neurodegenerative diseases [100, 179].

### Fyn signaling in astrocytes and synapse–glia interactions

Astrocytes belong to the “tripartite synapse” part, with functions beyond passive support. They regulate the construction of the synaptic microenvironment [180, 181]. Specifically, astrocytes can release ATP via connexin/pannexin-related channels. The ATP undergoes extracellular metabolism to form adenosine, which precisely regulates the strength and the plasticity of synaptic transmission [182, 183]. Astrocytes also rapidly clear excessive glutamate from the synaptic cleft via glutamate-glutamine cycling and high-affinity glutamate transport systems to limit excitatory signal spillover and reduce excitotoxicity risks [184, 185]. Furthermore, the Ca^2+^ waves between astrocytes coordinate local neuronal activity [186]. Meanwhile, the vascular end-feet of astrocytes mediate glucose uptake, glycogen mobilization, and lactate delivery, thereby providing critical energy support for high-metabolic-load synapses. Consequently, this maintains synaptic homeostasis and neural network efficiency [187–189].

In the context of neurodegeneration, SFKs, such as Fyn, can be up-regulated or abnormally activated in glial cells, accompanied by glial reactivity and inflammation-related phenotypes [51, 173]. Following these inflammatory changes, astrocytic Fyn signaling emerges as a critical regulator of prolonged neuroinflammation and synapse–glia crosstalk. Specifically, long-term exposure to inflammatory stimuli, such as TNF, selectively upregulates the FynT isoform in astrocytes [173]. The specific activity of astrocytic FynT kinase promotes persistent inflammatory responses, resulting in substantial induction of pro-inflammatory cytokines (e.g., IL-1β and IL-6) via activation of the NF-κB and PKCδ pathways within astrocytes. Additionally, the NF-κB and PKCδ pathways impair the glial clearance of glutamate via glutamate transporter 1 (GLT-1), a member of the excitatory amino acid transporters (EAATs) family. As a result, this impairment leads to neuronal hyperexcitability and worsened excitotoxicity **(**Fig. 3**)** [173, 190–192]. Conversely, Fyn also acts as a complex modulator of oxidative stress in the synaptic microenvironment. For instance, Fyn regulates the expression of inducible nitric oxide synthase (iNOS) in stimulated astrocytes via modulation of ERK (extracellular signal-regulated kinase) phosphorylation [193]. Remarkably, deficiency in Fyn actually promotes the production of iNOS and neurotoxic nitric oxide, suggesting its strict regulatory control over glial activation [193]. Within the context of neurodegenerative pathologies, such as AD, the overexpression of Fyn in neural networks exposed to Aβ directly triggers an astroglial injury response, characterized by prominent reactive astrocytosis [194]. Disrupting this pathological synapse–glia interaction through pharmacological inhibition of Fyn not only restores the existing synapse loss and memory deficits but also substantially reduces microglial activation [141], highlighting the central role of Fyn in coordinating synaptic and glial homeostasis.

### Coupling of neuroinflammation and tau pathology via Fyn

Mounting evidence suggests that neuroinflammation and tau pathology are not independent processes, but are coupled and form feed-forward amplification loops along the Fyn-mediated signaling axis [51, 195]. As detailed previously, pathological stimuli (e.g., LPS or TNF-α) drive microglial Fyn activation, which triggers the PKCδ/MAPK/NF-κB cascade to establish a sustained inflammatory microenvironment [100, 142]. In this context, inflammasome activation leads to the release of IL-1β, and meanwhile glial cells secrete pro-inflammatory cytokines like IL-6. Both IL-1β and IL-6 directly exacerbate aberrant tau phosphorylation. Ultimately, these responses promote the intracerebral spread of phosphorylated tau [196–200]. Conversely, pathological tau can also activate microglia and reinforce inflammatory output, thereby forming a self-sustaining vicious cycle **(**Fig. 3**)** [197, 201]. Thus, Fyn can be regarded as a key molecular bridge connecting neuroinflammation and tau pathology. Moreover, it serves as a significant potential intervention point for disrupting this positive feedback network and delaying disease progression [100].

### Cell-type-specific roles of Fyn in disease progression

Fyn exhibits differences in expression level and subcellular localization across cell types, which determine its functional roles and pathological importance in disease [51, 89, 202]. Within neurons, Fyn is primarily localized within synaptic signaling microdomains, where it regulates signaling through excitatory and inhibitory receptors (e.g., the NMDAR and GABA_A_ receptor complexes); upon coupling with tau protein and co-exporting to the PSD, Fyn modulates neuronal sensitivity to excessive glutamatergic receptor activation and neurotoxic stimuli [52, 70, 203]. In glial cells, especially microglia, Fyn tends to act as an inflammation amplification node. Upon pathological stimulation, its expression is upregulated, driving activation of the PKCδ–MAPK/NF-κB axis, facilitating pro-inflammatory effects and oxidative/nitrosative stress responses, and accelerating neural circuit damage [100, 204, 205]. Therefore, a comprehensive understanding of the cell type-specific Fyn signaling networks is a crucial prerequisite for targeted interventions to attain an equilibrium between “efficacy and safety” [68, 89].

## Fyn across the neurodegenerative disease spectra

Although the Fyn–tau axis is most extensively studied in AD, evidence indicates that the Fyn-mediated signaling abnormalities are not limited to a single disease type [3, 89]. In different primary tauopathies and other neurodegenerative diseases, Fyn may contribute to synaptic dysfunction and inflammatory responses through different mechanisms [36, 51, 100]. Therefore, Fyn may act as a shared molecular pathway connecting different pathological features. This may also explain why many neurodegenerative diseases have overlapping mechanisms [29, 89].

### AD

In AD, Fyn is a key molecule for Aβo-triggered postsynaptic toxic signaling. In dendritic spines and PSD, Fyn phosphorylates tyrosine residues on NMDAR subunits, thereby enhancing glutamatergic signaling [3, 25, 29]. Fyn and tau form a functional complex, which causes abnormal phosphorylation of tau, and tau accumulation at atypical postsynaptic sites. These changes block synaptic transmission and plasticity, and are closely related to memory loss and cognitive decline [3, 25, 29].

Studies have shown that the Fyn–tau axis is the key to early network hyperexcitability and synaptic toxicity amplification in AD [194, 206]. Decreasing the expression of Fyn/tau will reduce the Aβo-related synaptic toxicity, and improve cognitive function. Excessive Fyn activity will aggravate synaptic damage and increase cognitive impairment. Reducing tau levels can alleviate these harmful effects [194, 206]. Targeted inhibition of Fyn can reduce inflammatory responses and improve cognitive deficits in preclinical AD models. These findings provide support for early clinical exploration. Collectively, Fyn is a promising therapeutic target in early-stage AD [68, 207].

### Frontotemporal dementia and primary tauopathies

Abnormal Fyn-related signaling has also been reported in different primary tauopathies. However, the underlying mechanisms may not be the same, because tau isoforms and pathological distribution patterns differ across diseases [25]. Current findings indicate that Fyn interacts directly with tau, and acts as a crucial molecular link that connects amyloid and tau pathologies in neurodegenerative conditions [19].

For instance, the postsynaptic accumulation of Fyn constitutes the biological foundation of synaptotoxicity in dementia disorders, including FTLD with tau pathology. The pathogenic sequestration of Fyn depends upon the capacity of P301L tau to assemble into biomolecular condensates, which underscores the essential role of LLPS in regulating nanocluster formation and subsequent intracellular signaling processes [113].

Padmanabhan et al. employed single-molecule tracking to analyze the nanoscale distribution of Fyn in mouse hippocampal neurons and altered tau expression. They found that tau regulates the nanoscale organization of Fyn in dendrites, and the pathological tau P301L mutation may contribute to synaptic dysfunction by inducing abnormal Fyn nanoclustering in spines [20].

Liu et al. generated somatic transgenic FTDP-17 tauopathy model with the presence or absence of Fyn. They found that Fyn deficiency reduces the P301L tau-induced NFTs and level of tau hyperphosphorylation at disease-related sites. Fyn depletion also reduces activation of neuroinflammatory cells caused by mutant tau. Furthermore, the P301L tau-induced behavioral abnormalities and synaptic protein depletion were not observed in the Fyn knockout tauopathy model. The results confirm that Fyn is a key protein in the pathogenesis of FTDP-17 [36]. Briner and colleagues further demonstrated that Fyn plays an indispensable role in mediating the formation of high-molecular-weight tau aggregates. By generating the Tg/Fyn^−/−^ transgenic mouse model that combines mutant tau overexpression and Fyn knockout, they established that Fyn drives tau pathology independently of Aβ [104].

Furthermore, the *MAPT* A152T mutation has been identified as a risk factor for frontotemporal dementia spectrum disorders [208–210]. Another study in human iPSC-derived neurons indicated that the Fyn–tau interaction is essential for modulating neuronal activity under physiological conditions, and the A152T mutation increases neuronal activity by reinforcing this intrinsic Fyn-tau interaction [211]. The Fyn–tau complex has increasingly become an attractive drug target for the treatment of tauopathies, and research on inhibitors targeting this interaction is already underway [103, 212, 213].

### PD

Although PD is characterized primarily by α-syn pathology, a growing body of experimental evidence underscores the critical role of aberrant Fyn signaling in its pathogenesis through multiple mechanisms, including α-syn phosphorylation, dopaminergic neuronal loss, and neuroinflammation [100, 214].

Striatal-enriched tyrosine phosphatase (STEP) is a protein tyrosine phosphatase that dephosphorylates a number of substrates, including Fyn. Parkin ubiquitinates STEP and thereby regulates its level through the proteasome system. Elevated STEP activity and the subsequent dysfunction of Fyn have been associated with the overexpression of PD risk genes, such as *PARK2*. In addition, STEP accumulation has been detected in the striatum of sporadic PD brains [215]. Soluble Aβ signalling has been shown to dephosphorylate STEP, upregulating its activity [216]. Considering the similarities in pathological protein accumulation between AD and PD, α-syn may interact in a manner similar to Aβ, upregulating STEP, reducing Fyn kinase activity, and enhancing GluN2B endocytosis [88].

Extracellular α-syn binds to membrane receptors, thereby triggering the activation of Fyn. Fyn then upregulates the activity of PKCδ, which in turn activates NF-κB and induces the translocation of its p65 component into the nucleus, resulting in the transcription of pro-inflammatory cytokine genes such as IL-1β. Simultaneously, the activation of Fyn leads to the internalization of α-syn aggregates, which primes the NLRP3 inflammasome both directly and indirectly (via mitochondrial and lysosomal dysfunction), leading to the activation and conversion of pro-IL-1β to IL-1β [168].

Furthermore, α-syn oligomers engage in an interaction with PrP^C^ within glutamatergic synapses. This interaction initiates a signaling cascade that encompasses the phosphorylation of Fyn kinase via mGluR5 and the activation of the GluN2B subunit of NMDARs, resulting in synaptic dysfunction [217]. Inactivation of PrP^C^ impedes the toxic effects of α-syn on synaptic function, which is attributed to the prevention of Fyn/GluN2B signaling and the re-establishment of Ca^2+^ homeostasis [218].

Following autophosphorylation, Fyn becomes activated and subsequently phosphorylates the tyrosine residue 125 within the C-terminal domain of both wild-type and mutant (A53T, A30P) α-syn [214]. The phosphorylation of tyrosine 125 of α-syn by Fyn might impede the interaction between α-syn and phosphoinositide-3 kinase enhancer L (PIKE-L). PIKE-L is a GTPase that is abundant in nerve termini and exerts neuroprotective effects through activation of phosphoinositide 3-kinase upon interaction with its receptors [219]. The binding between α-syn and PIKE-L has been demonstrated to be serine 129-dependent. Moreover, the interaction between α-syn and PIKE-L may result in the sequestration of PIKE-L into Lewy bodies, followed by overactivation of AMPK and death of dopaminergic neurons [219].

Another study indicated that the phosphorylation of α-syn at tyrosine 125 by Fyn might cause the dissociation of α-syn from TrkB receptors, possibly resulting in neuroprotection [220]. Unphosphorylated α-syn can directly bind to the catalytic domain of TrkB receptors, inhibiting their distribution in lipid rafts, and suppressing BDNF/TrkB signaling, which ultimately leads to increased dopaminergic neuronal death [220].

### ALS and other neurodegenerative disorders

Research on ALS and other neurodegenerative diseases has also reported abnormal Fyn-related signals, especially in superoxide dismutase 1 (SOD1) mutant mouse models. In the motor cortex and spinal cord of SOD1^G93A^ mice, the activity of Fyn kinase is notably up-regulated. This increase leads to excessive tyrosine phosphorylation of the NMDAR subunit GluN2B, which further worsens excitotoxicity and motor neuron death [221]. In addition, during the neuroinflammatory process of ALS, Fyn works with other kinases to promote immune activation and mast cell differentiation. Therefore, broad-spectrum tyrosine kinase inhibitors that target Fyn, such as masitinib, and microglial regulators such as colony-stimulating factor 1 receptor, have shown potential to reduce neuroinflammation and prolong survival in disease models [222].

## Therapeutic targeting of Fyn: strategies, evidence, and challenges

Given the role of Fyn in several disease-related pathways, its potential as a therapeutic target has received increasing attention. Among SFK members, Fyn has the most direct connection to tau phosphorylation, postsynaptic localization, PSD-95/NMDAR coupling and AD-related synaptotoxicity, and its functions are only partially compensated by Lck, Lyn, Yes, or other SFK members. Currently, both direct small-molecule inhibitors of Fyn and indirect strategies regulating its activity have shown beneficial effects in reducing synaptic damage and ameliorating cognitive impairment in experimental models [141, 207]. However, Fyn also plays important roles under normal physiological conditions. Therefore, how to suppress its pathological activity without causing adverse effects remains a major challenge in translational research [89].

### Small-molecule Fyn inhibitors: preclinical and clinical evidence

Studies in multiple experimental models suggest that small-molecule Fyn inhibitors may improve synaptic and cognitive functions in CNS diseases [68, 194]. There are certain compounds at preclinical stage, such as certain flavonoids, which can inhibit Fyn activity at low micromolar concentrations [223]. These inhibitors bind to the ATP-binding pocket of Fyn and form hydrogen bonds with the activation loop, the αC-helix and the gatekeeping residue Thr342, maintaining strong binding affinity and stability [223]. By mitigating the neurotoxic effects of Aβ and tau and improving neuronal function, these compounds exhibit promising therapeutic potential in neurodegenerative diseases such as AD [141]. Many inhibitors originally developed for cancer may be tested for neurodegenerative diseases. Some small-molecule inhibitors have entered clinical trials, providing important proof of concept for Fyn-targeted therapy [207].

Saracatinib (AZD0530) is a quinzoline-based SFK inhibitor. It suppresses Src, Lyn, Yes, and Fyn [224]. Despite the fact that saracatinib exhibited limited efficacy in cancer treatment, it shows fine blood–brain barrier penetration and favorable pharmacokinetic properties. Because of this, researchers have considered its application in CNS disorders [225]. Preclinical studies suggest that saracatinib might hold potential for the treatment of tauopathies [226, 227]. However, recent trials in patients with mild to moderate AD found no major benefits, with no significant effect on clinical efficacy measures or regional cerebral glucose metabolism [207]. Masitinib is a phenylaminothiazole derivative that is under research for AD treatment. Inhibiting Fyn may reduce excessive phosphorylation of tau protein and slow the progression of NFTs. Clinical studies have shown that masitinib treatment may slow the rate of cognitive decline in AD [228]. Dasatinib is an SFK inhibitor that was demonstrated to regulate microglial activation and improve the memory of transgenic AD mice [228].

### Combination therapies targeting the Fyn-centered signaling axes

Combination therapy can improve the efficacy and reduce the risk of side effects [229]. Modulating Fyn and key nodes can prevent the amplification of pathological signals while maintaining the basic functions [68]. Most reported Fyn inhibitors are ATP-competitive [230]. However, due to the lack of selectivity, innovative multi-target approaches are needed for better safety and maintenance of physiological pathways such as myelination [228]. As evidenced by the selective Fyn inhibitor saracatinib, which failed to significantly slow the decline in cerebral metabolic rate for glucose or clinical cognitive symptoms in mild AD patients due to the dose-limiting tolerability issues preventing sufficient brain target engagement, single-target approaches often fail to alter the progression of multifactorial neurodegenerative diseases [231, 232]. Consequently, multi-target intervention strategies are under the development [232].

Specifically, dual-target inhibitors that simultaneously modulate Fyn and other key kinases, such as glycogen synthase kinase-3β (GSK-3β), have emerged as a promising avenue to achieve comprehensive neuromodulation and neuroprotection [232]. The abnormal activation of both Fyn and GSK-3β is involved in neurodegenerative disorders including AD and PD [233, 234]. Therefore, both kinases are essential therapeutic targets for dual-modulation strategies [235, 236]. For instance, ARN25068, a well-balanced dual inhibitor of GSK-3β and Fyn [237], also shows inhibitory activity against dual-specificity tyrosine-phosphorylation-regulated kinase 1A (DYRK1A), another target in AD and tauopathies. In the Tau0N4R-TM-tGFP U2OS cell line, ARN25068 reduces tau phosphorylation, suggesting promising potential for developing comprehensive triple GSK-3β/Fyn/DYRK1A inhibitors to resist tau hyperphosphorylation [228]. Similarly, following an extensive structure–activity relationship (SAR) screening, a recent investigation reported a series of azaindole-based dual-selective nanomolar inhibitors, identifying compound 43 as the lead compound [232]. Ultimately, the design of such multitarget inhibitors is a promising yet relatively under-investigated therapeutic strategy.

### Safety, specificity, and translational challenges

Fyn presents a challenging therapeutic target due to its widespread expression and high homology with other SFKs, which frequently leads to unintended off-target effects [228]. Because the kinase domains of SFK members are highly conserved, it is generally difficult to achieve complete specificity for Fyn alone. Even so, several well-known drugs show some degree of selective activity toward different SFK members, including Fyn. In addition, some inhibitors that were first developed for non-CNS diseases have later been tested in CNS disorders. This repurposing of non‑CNS drugs for CNS disorders was partly due to the high similarity of FynB and FynT catalytic domains [238]. Notably, Lee et al. reported that the selective increase of FynT is linked to reactive astrogliosis and NFTs in AD, and that FynT has higher kinase activity than FynB [50, 51]. However, no isoform-specific inhibitor that selectively targets FynT has been developed so far.

This limited selectivity can directly affect clinical outcomes. For example, saracatinib inhibits both Src and Fyn, and thus may cause off-target effects that weaken its therapeutic value [228]. In contrast, masitinib has shown better safety, efficacy, and tolerability than saracatinib in patients with mild-to-moderate AD. This makes it a more promising option and may also improve patient adherence [239]. Dasatinib also stands out as a potential candidate for AD treatment. It strongly inhibits Fyn, and has shown a safety profile from its clinical use in leukemia [89]. Overall, Fyn inhibitors have potential to attenuate synaptic dysfunction and cognitive decline. However, more studies and clinical trials are needed to fully determine their efficacy and safety in neurodegenerative diseases.

## Conclusions and future perspectives

Fyn is not only a central link between tau pathology, Aβ toxicity, and synaptic failure, but also a direct driver of synaptic damage. Evidence from the past two decades shows that the move from mechanistic research to clinical treatment is far more complex than expected by simply regarding Fyn as a pathway that can be controlled by a basic “on/off” strategy. It is imperative to elucidate the fine structural dynamics of Fyn in LLPS. Moreover, it is essential to formulate strategies for differentiating between “physiological LTP signals” and “pathological toxicity coupling” in order to bridge the disparity between mouse models and human patients.

## Data Availability

No datasets were generated or analysed during the current study.

## Abbreviations

AD: Alzheimer’s disease
ALS: Amyotrophic lateral sclerosis
AMPK: AMP-activated protein kinase
Aβ: Amyloid-β
Aβo: Aβ oligomers
BDNF: Brain-derived neurotrophic factor
CaMKII: Calcium/calmodulin-dependent protein kinase II alpha
CDK5: Cyclin-dependent kinase 5
CNS: Central nervous system
CSF: Cerebrospinal fluid
DYRK1A: Dual specificity tyrosine-phosphorylation-regulated kinase 1A
EAATs: Excitatory amino acid transporters
ER: Endoplasmic reticulum
ERAD: Endoplasmic reticulum-associated degradation
FTDP-17: Frontotemporal dementia and Parkinsonism associated with chromosome 17
FynB: Brain-type isoform of Fyn
FynT: T-cell-type isoform of Fyn
GABA_A_: Gamma-aminobutyric acid type A
GLT-1: Glutamate transporter 1
GluN2A: Glutamate ionotropic receptor NMDA type subunit 2A
GluN2B: Glutamate ionotropic receptor NMDA type subunit 2B
GSK-3β: Glycogen synthase kinase-3β
GWAS: Genome-wide association studies
IL: Interleukin
iNOS: Inducible nitric oxide synthase
LLPS: Liquid-liquid phase separation
LPS: Lipopolysaccharide
LTP: Long-term potentiation
MAPK: Mitogen-activated protein kinase
mGluR5: Metabotropic glutamate receptor 5
NFTs: Neurofibrillary tangles
NF-κB: Nuclear factor kappa B
NLRP3: NOD-like receptor family pyrin domain-containing 3
NMDA: N-methyl-D-aspartate
NMDAR: N-methyl-D-aspartate receptor
OLs: Oligodendrocytes
OPC: Oligodendrocyte precursor cell
PD: Parkinson’s disease
PIKE-L: Phosphoinositide-3 kinase enhancer L
PKCδ: Protein kinase Cδ
PrP^C^: Cellular prion protein
PSD: Postsynaptic density
PSD-95: Postsynaptic density protein 95
PXXP: Proline-X-X-proline
ROS: Reactive oxygen species
SAP102: Synapse-associated protein 102
SAP97: Synapse-associated protein 97
Sema3A: Semaphorin-3A
SFK: Src family kinase
SOD1: Superoxide dismutase 1
STEP: Striatal-enriched tyrosine phosphatase
TNF-α: Tumor necrosis factor-α
TrkB: Tropomyosin receptor kinase B
UPR: Unfolded protein response
α-syn: α-Synuclein
